# Revisiting the Formation and Tunable Dissociation of a [2]Pseudorotaxane Formed by Slippage Approach

**DOI:** 10.3390/ijms16048254

**Published:** 2015-04-13

**Authors:** Ken Cham-Fai Leung, Kwun-Ngai Lau, Wing-Yan Wong

**Affiliations:** 1 Department of Chemistry and Institute of Creativity, The Hong Kong Baptist University, Kowloon Tong, Kowloon, Hong Kong, China; 2 Institute of Molecular Functional Materials, University Grants Committee, Hong Kong, China; 3 Department of Chemistry, The Chinese University of Hong Kong, Shatin, NT, Hong Kong, China; E-Mails: terrylau1036@gmail.com (K.-N.L.); Wingwong.wwy@gmail.com (W.-Y.W.)

**Keywords:** rotaxane, thermodynamic synthesis, dissociation, slippage, crown ether

## Abstract

A new [2]pseudorotaxane **DB24C8**⊃**1**-H·PF_6_ with dibenzo[24]crown-8 (**DB24C8**) crown ether-dibenzylammonium (**1**-H·PF_6_) binding which was formed by slippage approach at different solvents and temperature, had been isolated and characterized by NMR spectroscopy and mass spectrometry. The [2]pseudorotaxane **DB24C8**⊃**1**-H·PF_6_ was stable at room temperature. The dissociation rate of [2]pseudorotaxane **DB24C8**⊃**1**-H·PF_6_ could be tuned by using different stimuli such as triethylamine (TEA)/diisopropylethylamine (DIPEA) and dimethyl sulfoxide (DMSO). In particular, the dissociation of [2]pseudorotaxane **DB24C8**⊃**1**-H·PF_6_ by an excess of TEA/DIPEA base mixture possessed a long and sustained, complete dissociation over 60 days. Other stimuli by DMSO possessed a relatively fast dissociation over 24 h.

## 1. Introduction

Rotaxanes contain a linear dumbbell-shaped component bearing bulky end-groups or stoppers around which one or more macrocycles are trapped. On the other hand, pseudorotaxanes are temporally encircled around an unstoppered thread through noncovalent interactions from which they are readily susceptible to dissociation without breaking a covalent bond [1,2,3,4]. Rotaxanes and pseudorotaxanes have been studied extensively for the ability of the interlocked ring to be switched on demand by external stimuli such as pH [5,6], electrochemical reagents [7], heat [8,9], moisture [10,11], salt [12], light [13], *etc.* Coupled with their ability to be customized and optimized for nanoscale functions, these interlocked molecules are excellent candidates as movable elements in the construction of nanovalves [14] based on a porous, solid-phase support, for controlled substrate release. Many examples have been demonstrated about their relatively fast substrate releases within 24 h by tuning the pH values in the solution [12]. Currently, no pseudorotaxane or rotaxane building blocks are available and suitable for the construction of vehicles for sustained substrate release over a week.

The construction of rotaxane-like assemblies has recently relied upon thermodynamic, templated reactions with enhanced efficiencies [15,16,17,18,19,20,21,22,23,24]. The term “slippage” has been coined [25,26,27,28] for pseudorotaxane synthesis that employs thermodynamic threading of macrocycle to a “dumbbell”. This strategy utilizes (1) the size complementarily between the macrocycle and the “dumbbell’s” stoppers; and (2) the stabilizing noncovalent bonding interactions between the macrocycle and the “dumbbell’s” rod. In this strategy, the macrocycle and the dumbbell have been separately synthesized, prior to heating them together in solution so that the free energy of activation for the thermodynamic threading (slippage) of macrocycle to dumbbell can be overcome. The presence of a template on the dumbbell’s rod renders the pseudorotaxane structure more stable so that the free energy of activation for its dissociation becomes insurmountable when the solution has been cooled to ambient temperature. There are several disadvantages of the slippage approach in terms of reaction time and stability. Generally, it requires a long reaction time with a large slippage rate (*k*_on_) for pseudorotaxane formation from usually a few days up to 90 days [27]. However, the dissociation of pseudorotaxane will sometimes have an uncontrollable, small slippage rate (*k*_off_). Therefore, there is a need to study a better reaction condition for pseudorotaxane formation by slippage and to tune the pseudorotaxane dissociation rate (*k*_off_ << *k*_on_) from days to hours.

## 2. Results and Discussion

**Figure 1:**
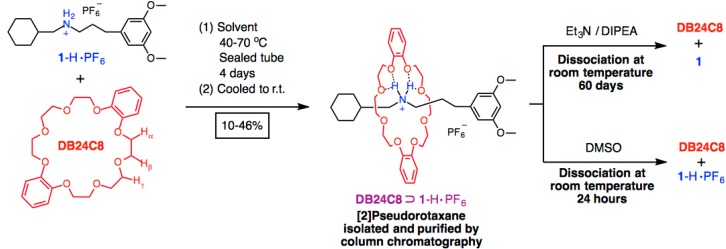
Molecular structures of the crown ether **DB24C8** and the ammonium thread “dumbbell” **1**-H·PF_6_. The 3,5-dimethoxyaryl group of the thread acts as an effective stopper to prevent the extrusion of macrocycle while the cyclohexyl group of the thread allows the slipping of macrocycle upon heating in selected solvents. The thermodynamically stable [2]pseudorotaxane **DB24C8**⊃**1**-H·PF_6_ was isolated and subjected to dissociation studies with excess amine base (Et_3_N and DIPEA) mixture and dimethyl sulfoxide (DMSO).

Herein, we employ a slippage approach in one pot (Figure 1) to yield a thermodynamically stable [2]pseudorotaxane [29,30] by mixing an ammonium thread (dumbbell) **1**-H·PF_6_ with dibenzo[24]crown-8 (**DB24C8**). Benzo-crown ether **DB24C8** is capable to recognize with secondary ammonium ions by virtue of [N^+^–H···O] and [N^+^–C–H···O] hydrogen bonds, electrostatic interactions, and augmented with some aromatic π-π interactions [31,32,33,34,35,36]. The 3,5-dimethoxyaryl moiety at one end of the dumbbell **1**-H·PF_6_, is a relatively bulky stopper that can effectively block the passageway of **DB24C8** at high reaction temperature. On the other hand, the cyclohexyl ring of the dumbbell-like thread, in contrast, allow the crown ether to thread through to the dumbbell’s rod at elevated reaction temperature with the molecular flipping of between their chair-boat-chair forms [31,32,33,34,35,36,37].

The reaction time of the pseudorotaxane synthesis was fixed in 4 days with reasonable yields (~50%). The percentage yields (Table 1) of individual [2]pseudorotaxane after a reaction time of 4 days, have been determined by ^1^H NMR spectroscopy according to their “bound” and “free” signal intensities [36,37,38]. From the results, the reaction yields and rates in synthesizing the [2]pseudorotaxanes are sensitive to different solvents and temperatures. In particular, the percentage yield of the [2]pseudorotaxane **DB24C8**⊃**1**-H·PF_6_ was found to be the highest (46%) after a reaction temperature at 70 °C with MeCN compared to using PhMe (70 °C) and CH_2_Cl_2_ (40 °C). This is partially because, in polar solvents, the [2]pseudorotaxane might undergo intra and/or intermolecular aggregation of the hydrophobic alkyl chains and this will overcome the extrusion effect. The [2]pseudorotaxane **DB24C8**⊃**1**-H·PF_6_ could be isolated by flash chromatography on silica gel (CH_2_Cl_2_/THF = 7/1) and characterized by NMR spectroscopy and mass spectrometry.

**Table 1:** Percentage yields of the [2]pseudorotaxanes after slippage reaction for 4 days. Analyzed by ^1^H NMR spectroscopy (400 MHz, 298 K).

Solvent (Reaction Temperature in Sealed Tube)	DB24C8⊃1-H·PF_6_
MeCN (70 °C)	46%
PhMe (70 °C)	30%
CH_2_Cl_2_ (40 °C)	10%

**Figure 2:**
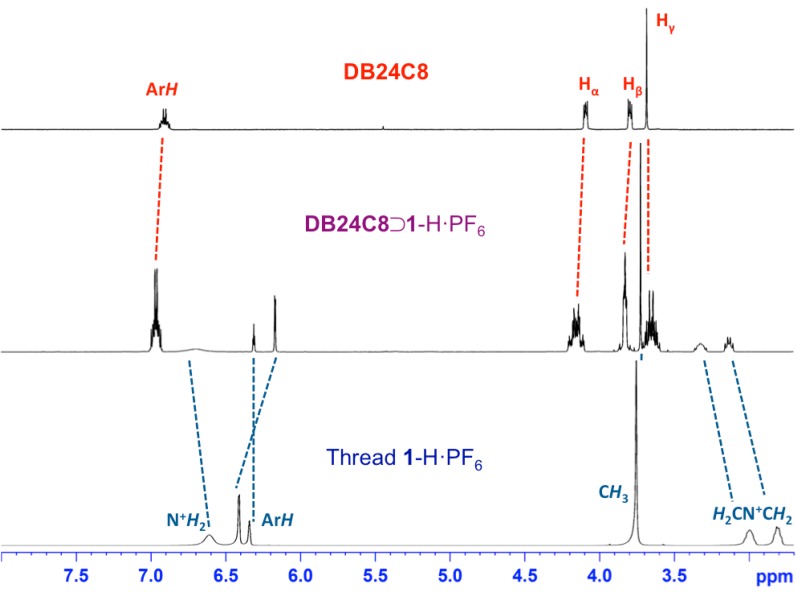
Partial ^1^H NMR spectra (400 MHz, CD_3_CN, 298 K) of crown ether **DB24C8**, isolated pure [2]pseudorotaxane **DB24C8**⊃**1**-H·PF_6_, and ammonium thread **1**-H·PF_6_.

^1^H NMR spectroscopy was employed to evaluate the structural features of [2]pseudorotaxanes. By way of an example, the isolated and pure [2]pseudorotaxane **DB24C8**⊃**1**-H·PF_6_ reveals (Figure 2) proton chemical shifts of the –C*H*_2_NH_2_^+^C*H*_2_– moieties at δ = 3.14 and 3.32 ppm (bound), comparing to the –C*H*_2_NH_2_^+^C*H*_2_– moieties of its thread **1**-H·PF_6_ at δ = 2.81 and 3.06 ppm (free).

**Figure 3:**
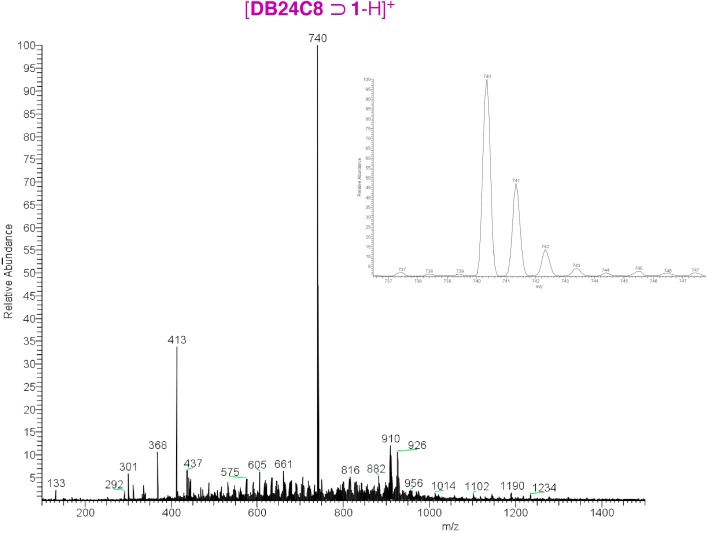
Electrospray ionization mass spectrometry (ESI-MS) of the [2]pseudorotaxane **DB24C8**⊃**1**-H·PF_6_, showing the molecular ion signal [M–PF_6_]^+^ at *m/z* 740.

High-resolution electrospray ionization mass spectrometry (ESI-MS) has been employed (Figure 3) to further characterize the [2]pseudorotaxane. The molecular ion peak at *m*/*z* 740 which is the most abundant peak in the spectrum, is corresponded to the [M–PF_6_]^+^ ion of the [2]pseudorotaxane.

**Figure 4:**
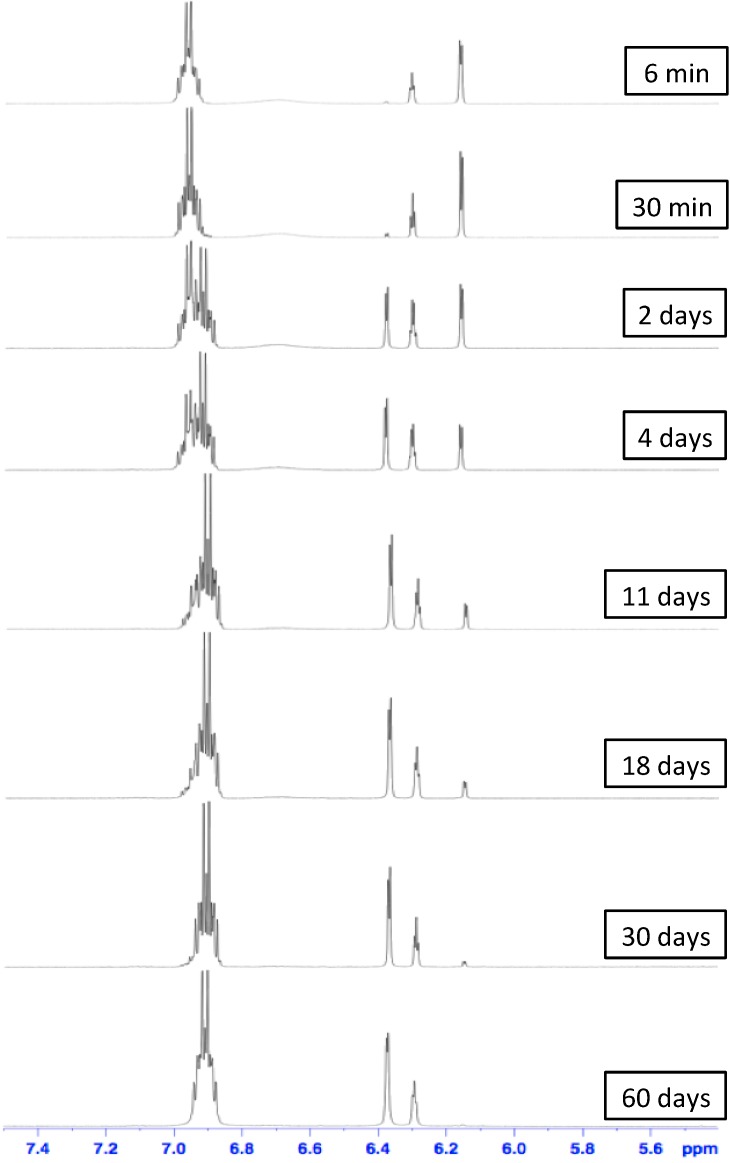
Partial ^1^H NMR spectra (400 MHz, CD_3_CN, 298 K) showing the extrusion of **DB24C8** macrocycle from the isolated [2]pseudorotaxane **DB24C8**⊃**1**-H·PF_6_ with excess triethylamine (TEA)/diisopropylethylamine (DIPEA) mixture in CD_3_CN over time.

Furthermore, the dissociation and stabilities of [2]pseudorotaxane towards organic amine bases and a hydrogen bond disrupting solvent were evaluated. In particular, pure [2]pseudorotaxane **DB24C8**⊃**1**-H·PF_6_ was dissolved in CD_3_CN and was treated with an excess of triethylamine (TEA)/diisopropylethylamine (DIPEA) mixture [5,6,12]. The ammonium ion of the thread **1**-H·PF_6_ could be successfully deprotonated by the bases wherein the **DB24C8** loss its binding affinity towards the deprotonated, amine thread **1**. Since the template effect is lost, the [2]pseudorotaxane is no longer stable whereas extrusion of macrocycle occurs with the molecular flipping of the cyclohexyl ring. This extrusion behavior was monitored (Figure 4) by observing a significant decrease of characteristic signal at δ = 6.16 ppm (bound Ar*H* of **1**-H·PF_6_) as well as an increase of signal at δ = 6.38 ppm (free Ar*H* of **1**) from their ^1^H NMR spectra over time. Interestingly, the [2]pseudorotaxane requires almost 60 days to dissociate completely into two separate components at ambient temperature. The half-life of dissociation was determined to be approximately 2 days (Figure 6).

**Figure 5:**
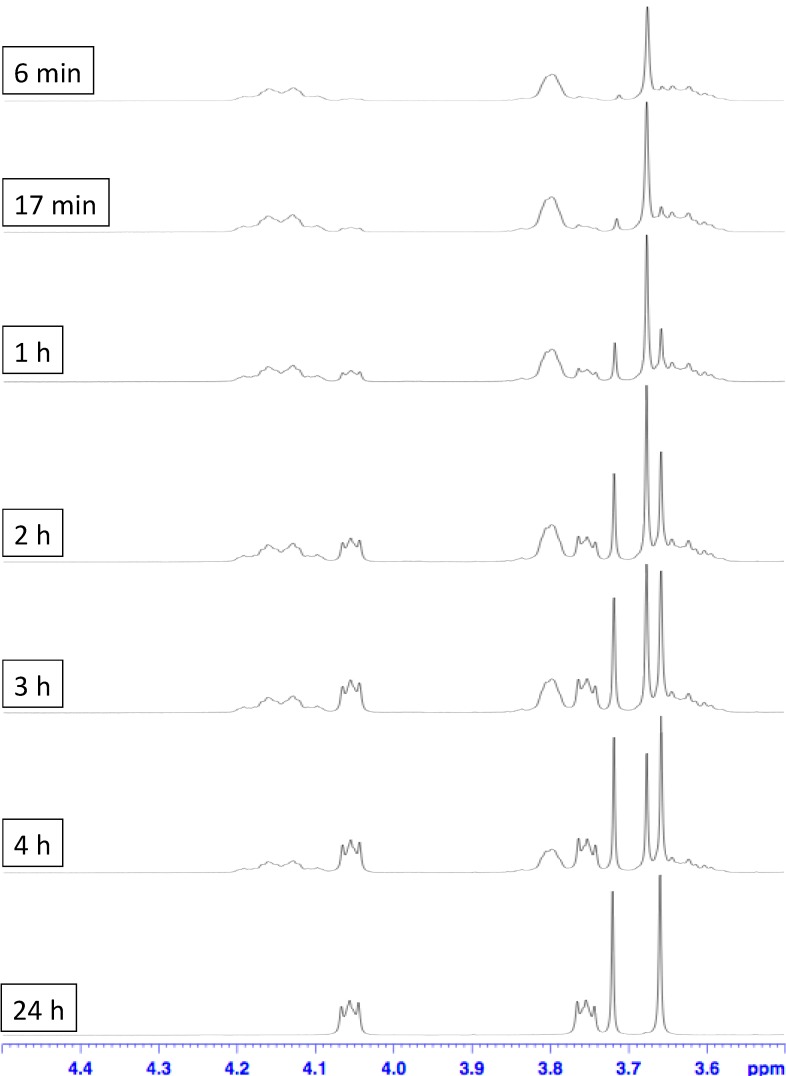
Stacked ^1^H NMR spectra (400 MHz, CD_3_SOCD_3_, 298 K) showing the extrusion of **DB24C8** macrocycle from the isolated [2]pseudorotaxane **DB24C8**⊃**1**-H·PF_6_ in DMSO-*d*_6_ over time.

**Figure 6:**
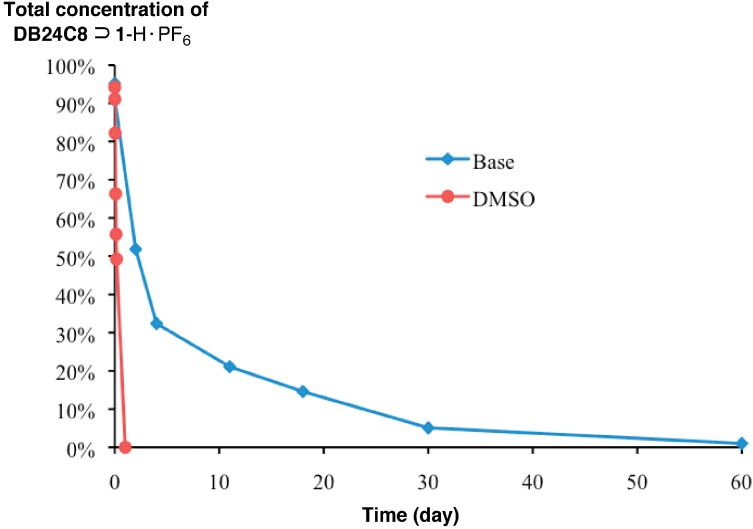
Dissociation data of the [2]pseudorotaxane **DB24C8**⊃**1**-H·PF_6_ in the presence of base and DMSO, characterized by ^1^H NMR spectroscopy. A plot of the concentration (%) of the [2]pseudorotaxane **DB24C8**⊃**1**-H·PF_6_ *versus* time (day).

On the other hand, by dissolving the pure [2]pseudorotaxane **DB24C8**⊃**1**-H·PF_6_ in a hydrogen bond disfavored solvent—dimethyl sulfoxide (DMSO) [1,27], extrusion of **DB24C8** from the thread **1**-H·PF_6_ occurred. Since the template effect is lost, the [2]pseudorotaxane is no longer stable whereas extrusion of macrocycle occurs with the molecular flipping of the cyclohexyl ring. This extrusion behavior was monitored (Figure 5) by observing the decreases of characteristic signals at δ = 3.81, and 4.14 ppm (bound –OC*H*_2_C*H*_2_O– of **DB24C8**) as well as the increases of signals at δ = 3.74, and 4.07 ppm (free –OC*H*_2_C*H*_2_O– of **DB24C8**) from their ^1^H NMR spectra (in DMSO-*d*_6_) over time. The [2]pseudorotaxane required only 24 h for a complete dissociation. The half-life of dissociation was determined to be approximately 4 h (Figure 6).

## 3. Experimental Section

**Scheme 1:**
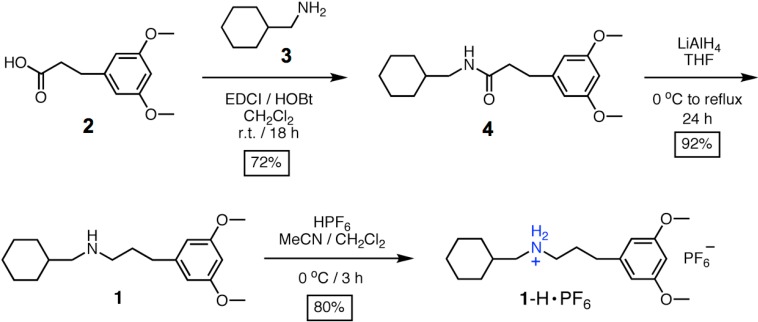
Synthesis of the ammonium thread **1**-H·PF_6_.

**General** **Information.** ^1^H NMR (400 MHz) and ^13^C NMR (100 MHz) spectra were recorded at room temperature in CDCl_3_ unless otherwise stated. Each solvent residual signal was used as the internal standard. Chemical shifts were reported as parts per million (ppm) in δ scale and coupling constants (*J*) were reported in hertz. Mass spectra were obtained on a double focusing sector mass spectrometer with electrospray ionization (ESI) technique. The reported molecular mass (*m*/*z*) values, unless otherwise specified, were mono-isotopic mass. All reactions were carried out under N_2_. All reactions were monitored by thin layer chromatography (TLC) performed on pre-coated silica gel 60 F_254_ plates, and compounds were visualized with a spray of 5% (*w*/*v*) dodecamolybdophosphoric acid in ethanol and subsequent heating. Flash chromatography was carried out on columns of silica gel (230–400 mesh). Tetrahydrofuran (THF) was freshly distilled prior to use from sodium/benzophenone ketyl under N_2_. CH_2_Cl_2_ was freshly distilled from CaH_2_. Cyclohexanemethylamine (**3**) and dibenzo[24]crown-8 (**DB24C8**) were commercially available from Sigma-Aldrich (St. Louis, MO, USA) while the starting compound (**2**) was synthesized according to the literature procedures [39]. The synthetic scheme of new compounds is shown in Scheme 1.

**Amide 4.** 1-[3-(Dimethylamino)propyl]-3-ethylcarbodiimide methiodide (EDCI, 2.53 g, 8.52 mmol) was added to a stirred solution which contained 3-(3,5-dimethoxyphenyl)-propionic acid (**2**, 1.43 g, 6.81 mmol), cyclohexanemethylamine (**3**, 1.00 mL, 7.69 mmol) and 1-hydroxybenzotriazole (HOBt) (1.14 g, 8.45 mmol) in CH_2_Cl_2_ (25 mL). The reaction mixture was stirred for 18 h at 25 °C and concentrated under reduced pressure. The residue was purified by flash chromatography on silica gel (hexane/EtOAc = 4/1) to afford the amide (**4**) (1.50 g, 72%) as a white solid. M.p.: 72.6–74.3 °C. *R*_f_: 0.58 (hexane/EtOAc = 4/1). ^1^H NMR: δ 0.64–0.81 (2 H, m, CH_2_), 0.92–1.14 (3 H, m), 1.20–1.34 (1 H, m), 1.43–1.62 (5 H, m), 2.38 (2 H, t, *J* = 7.6, CH_2_C*H*_2_), 2.77 (2 H, t, *J* = 7.6, CH_2_C=O), 2.92 (2 H, t, *J* = 6.4, CHC*H*_2_), 3.60 (6 H, s, CH_3_), 6.16 (1 H, s, Ar*H*), 6.23 (2 H, s, Ar*H*), 6.61 (1 H, t, *J* = 5.2, NH). ^13^C NMR: δ 25.5, 26.1, 30.5, 31.8, 37.57, 37.63, 45.4, 54.7, 97.6, 106.0, 143.1, 160.5, 172.1. ESI-MS: *m*/*z* 328 ([M + Na]^+^, 100%). HRESI-MS: calcd *m*/*z* for C_18_H_27_NO_3_Na: 328.1883, found: 328.1883 ([M + Na]^+^, 100%).

**Amine 1.** Lithium aluminum hydride (LAH) (0.77 g, 20.3 mmol) was added slowly to a stirred solution of amide (**4**) (1.43 g, 4.68 mmol) in THF (80 mL) at 0 °C. The solution was then heated to reflux for 24 h. The solution was cooled down to room temperature and then poured into an ice-water mixture. The resultant mixture was then extracted with CH_2_Cl_2_ (50 mL × 3) and the combined extracts were washed with brine, dried (MgSO_4_), filtered and evaporated *in vacuo* to give the crude product which was purified by flash chromatography on silica gel (hexane/EtOAc/Et_3_N = 240/120/1) to afford the amine (**1**) (1.25 g, 92%) as a colorless liquid. *R*_f_: 0.35 (hexane/EtOAc/Et_3_N = 240/120/1). ^1^H NMR: δ 0.73–0.88 (2 H, m, CH_2_), 0.99–1.23 (3 H, m), 1.28–1.42 (1 H, m), 1.50–1.83 (8 H, m), 2.34 (2 H, d, *J* = 6.8, CHC*H*_2_NH), 2.45–2.58 (4 H, m, ArC*H*_2_CH_2_ and ArCH_2_C*H*_2_), 3.65 (6 H, s, CH_3_), 6.20 (1 H, d, *J* = 2.0, Ar*H*), 6.26 (2 H, d, *J* = 2.0, Ar*H*). ^13^C NMR: δ 25.8, 26.4, 31.1, 31.2, 33.7, 37.6, 49.3, 54.8, 56.4, 97.4, 106.1, 144.2, 160.5. ESI-MS: *m*/*z* 292 ([M + H]^+^, 100%). HRESI-MS: calcd *m*/*z* for C_18_H_30_NO_2_: 292.2271, found: 292.2276 ([M + H]^+^, 100%).

**Ammonium salt 1-H·PF_6_.** To a stirred solution of amine (**1**) (1.25 g, 4.29 mmol) in a solvent mixture of CH_3_CN/CH_2_Cl_2_ (3:1) (20 mL), excess hexafluorophosphoric acid (HPF_6_) (1.20 mL, 8.73 mmol) was added dropwise at 0 °C. The reaction mixture was stirred for 3 h and then water (12 mL) was added to the mixture. The mixture was concentrated under reduced pressure and then extracted with CH_2_Cl_2_ (50 mL × 3). The combined extracts were washed with brine, dried (MgSO_4_), filtered and evaporated *in vacuo* to afford the ammonium salt **1**-H·PF_6_ (1.50 g, 80%) as a white solid. M.p.: 173.6–174.8 °C. ^1^H NMR (CD_3_CN): δ 0.87–1.07 (2 H, m, CH_2_), 1.08–1.34 (3 H, m), 1.57–1.82 (6 H, m), 1.93–2.12 (2 H, m, CH_2_), 2.61 (2 H, t, *J* = 7.6, ArC*H*_2_), 2.75–2.90 (2 H, m, CHC*H*_2_NH_2_), 2.93–3.10 (2 H, m, CH_2_C*H*_2_NH_2_), 3.76 (6 H, s, CH_3_), 6.34 (1 H, s, Ar*H*), 6.41 (2 H, s, Ar*H*), 6.61 (2 H, br s, NH_2_). ^13^C NMR (CD_3_CN): δ 26.5, 26.8, 28.1, 30.9, 33.4, 35.8, 49.4, 55.2, 56.0, 99.1, 107.5, 144.1, 162.2. ESI-MS: *m*/*z* 292 ([M–PF_6_]^+^, 100%). HRESI-MS: calcd *m*/*z* for C_18_H_30_NO_2_: 292.2271, found: 292.2265 ([M–PF_6_]^+^, 100%).

**Typical Synthesis of [2]Pseudorotaxane.** A solution of **1**-H·PF_6_ (17.5 mg, 0.04 mmol) and **DB24C8** (35.9 mg, 0.08 mmol) in a typical solvent (1.5 mL) was heated at 40 or 70 °C in a sealed tube for 4 days and then concentrated *in vacuo*. For an alternative method, a solution of **1**-H·PF_6_ (120 mg, 0.27 mmol) and **DB24C8** (40 mg, 0.089 mmol) in MeCN (3 mL) was heated at 70 °C in a sealed tube for 4 days and then concentrated *in vacuo*. The resulting residue was purified by flash chromatography on silica gel (CH_2_Cl_2_/THF = 7/1) to afford the [2]pseudorotaxane **DB24C8**⊃**1**-H·PF_6_ (34–36 mg, 46%) as a white solid. M.p. was not determined due to thermal instability. *R*_f_: 0.65 (CH_2_Cl_2_/THF = 7/1). ^1^H NMR (CD_3_CN): δ 0.56–0.72 (2 H, m, CH_2_), 0.78–0.93 (3 H, m), 1.40–1.55 (6 H, m, CH_2_), 1.79–1.91 (2 H, m, CH_2_), 2.41 (2 H, t, *J* = 7.2, ArC*H*_2_), 3.08–3.18 (2 H, m, CHC*H*_2_NH_2_), 3.27–3.38 (2 H, m, CH_2_C*H*_2_NH_2_), 3.58–3.70 (8 H, m, CH_2_CH_2_OC*H*_2_), 3.72 (6 H, s, CH_3_), 3.78–3.89 (8 H, m, ArOCH_2_C*H*_2_), 4.08–4.23 (8 H, m, ArOC*H*_2_CH_2_), 6.16 (2 H, d, *J* = 2.0, Ar*H*), 6.31 (2 H, d, *J* = 2.0, Ar*H*), 6.55–6.84 (2 H, br s, NH_2_), 6.90–7.02 (8 H, m, catechol Ar*H*). ^13^C NMR (CD_3_CN): δ 26.1, 26.5, 28.7, 30.9, 33.4, 36.3, 49.2, 55.4, 55.9, 69.0, 71.3, 71.9, 98.7, 107.4, 113.6, 122.4, 144.0, 148.5, 162.0. ESI-MS: *m*/*z* 740 ([M–PF_6_]^+^, 100%). HRESI-MS: calcd *m*/*z* for C_42_H_62_NO_10_: 740.4368, found: 740.4359 ([M–PF_6_]^+^, 100%).

**Typical [2]Pseudorotaxane Dissociation.** For dissociation with bases, [2]pseudorotaxane **DB24C8**⊃**1**-H·PF_6_ (5 mg) was dissolved in CD_3_CN (0.7 mL) in a NMR tube followed by the addition of 3 drops of Et_3_N (TEA) and 3 drops of *^i^*Pr_2_EtN (DIPEA) to the solution. ^1^H NMR spectra were recorded over time at 298 K. For dissociation with solvent, [2]pseudorotaxane **DB24C8**⊃**1**-H·PF_6_ (5 mg) was dissolved in CD_3_SOCD_3_ (0.7 mL) in a NMR tube. ^1^H NMR spectra were recorded over time at 298 K.

## 4. Conclusions

In summary, a [2]pseudorotaxane **DB24C8**⊃**1**-H·PF_6_ which was synthesized and isolated from a slippage approach, was found to be stable at room temperature. The [2]pseudorotaxane **DB24C8**⊃**1**-H·PF_6_ was characterized by NMR spectroscopy and mass spectrometry. The dissociation rate of [2]pseudorotaxane **DB24C8**⊃**1**-H·PF_6_ could be tuned by amine bases and DMSO solvent at room temperature from which the supramolecular interactions between the crown ether **DB24C8** and the ammonium can be tuned. When the crown ether **DB24C8** was lost its noncovalent interactions with the ammonium/amine, it could be detached from the dumbbell **1**-H·PF_6_ via the molecular flipping of between the chair-boat-chair forms of the cyclohexyl ring. In particular, the dissociation of [2]pseudorotaxane **DB24C8**⊃**1**-H·PF_6_ with amine bases possessed a long and sustained, complete dissociation over 60 days. The use of DMSO possessed relatively fast and complete dissociation (24 h). The selectively tunable dissociation rates by potentially varying the ratios of stimuli for macrocycle extrusion from the novel [2]pseudorotaxane provide avenues for constructing novel functional nanovalves that sustained release substrates for a much longer period of time.
